# Choroid plexus tumors in adults: a retrospective mono-institutional study

**DOI:** 10.1007/s10072-024-07894-x

**Published:** 2024-12-02

**Authors:** Elena Anghileri, Paola Gaviani, Anna Amato, Bianca Pollo, Rosina Paterra, Marcello Marchetti, Fabio M. Doniselli, Francesco Restelli, Marica Eoli, Ludmila de Oliveira Muniz Koch, Veronica Redaelli, Andrea Giorgio Botturi, Francesco DiMeco, Paolo Ferroli, Mariangela Farinotti, Antonio Silvani

**Affiliations:** 1 Neuroncology Unit, Fondazione IRCCS Istituto Neurologico Carlo Besta, Milan, Italy; 2 Department of Medicine and Surgery, University of Milano-Bicocca, Milan, Italy; 3 Neuropathology Unit, Fondazione IRCCS Istituto Neurologico Carlo Besta, Milan, Italy; 4 Neuroradiotherapy Unit, Fondazione IRCCS Istituto Neurologico Carlo Besta, Milan, Italy; 5 Neuroradiology Unit, Fondazione IRCCS Istituto Neurologico Carlo Besta, Milan, Italy; 6 Latin American Cooperative Oncology Group, Porto Alegre, Hospital Israelita Albert Einstein, São Paulo, Brazil; 7 Neurosurgery Department, Fondazione IRCCS Istituto Neurologico Carlo Besta, Milan, Italy; 8 Cancer Registry, Fondazione IRCCS Istituto Neurologico Carlo Besta, Milan, Italy

**Keywords:** Choroid plexus tumors (CPT), Choroid plexus neoplasms, TERT promoter mutation, Adult, Prognosis, Therapeutics

## Abstract

**Purpose:**

Choroid plexus tumors (CPT) are rare entities, and even rarer in adulthood.

**Methods:**

A retrospective consecutive series of 24 adult CPT patients was reviewed.

**Results:**

We described 24 adult CPTs. Clinical onset included cerebellar signs (*n* = 11, 45.8%), intracranial hypertension signs (*n* = 8, 33.4%), cranial nerves impairment (*n* = 5, 20.8%), incidental findings (*n* = 4, 16.6%), seizures (*n* = 1, 4.2%), spinal signs (*n* = 1, 4.2%). At first diagnosis, CPT was mostly located in the ventricular system, but other locations can occur, including the spine (one case); meningeal involvement was present in one, pre-surgical hydrocephalus in one case only. CPT histological grade ranged from grade 1 (*n* = 17), grade 2 (*n* = 6), and grade 3 (*n* = 1). TERTp mutation was detected in 17.6% (*n* = 3/17). TP53 mutation in 5.9% (*n* = 1/17). Gross Total, Subtotal, Partial resection and Biopsy were achieved in 17 (70.8%), 3 (12.5%), 3 (12.5%) and 1 (4.2%) of patients, respectively. 76% of cases (*n* = 16/21) experienced clinical worsening suddenly after surgery for different reasons, and mostly gradually recovered. For three cases no data was available. Adjuvant therapy was performed only for grades 2 and 3. At recurrence, surgery, radiosurgery, radiotherapy and chemotherapy were considered. The median Overall Survival from surgery was 219.25 months (95% CI, 188.83–249.67).

**Conclusions:**

We confirm that CPT can occur in adults and are mostly grade 1 tumors located in the ventricular system. The surgical approach is the gold standard, although 76% of clinical worsening occurred, often transient. Adjuvant treatment was limited to higher grade CPT; however, no consensus has already been achieved about adjuvant therapy.

## Introduction

Choroid plexus tumors (CPT) are rare entities, that account for up to 20% of brain tumors in children under the age of 1-year-old [1]. The overall age-adjusted incidence rate is 0.048 per 100 000; for adulthood 0.002 per 100 000 was reported [2]. From the year 2000 to 2019, 679 patients with CPT were reported from the Surveillance, Epidemiology, and End Result-SEER database, and 54.6% were pediatric [3].

CPT are mostly intraventricular neoplasms, subclassified into three gradings: grade 1 [(choroid plexus papilloma (CPP)], grade 2 [atypical (aCPP)] and grade 3 [choroid plexus carcinoma (CPC)] [4]. CPC may be associated with Li-Fraumeni syndrome, a cancer susceptibility syndrome caused by germline mutations of the *TP53* tumor suppressor gene [5].

Symptoms of CPT at onset are often associated with hydrocephalus and increased intracranial pressure, secondary to a rise in the production of cerebrospinal fluid (CSF) by the tumor or to mass effect [6, 7]. Few patients have lateralizing signs [8].

Patients harboring CPP usually experience good long-term outcomes if gross total surgical resection can be achieved, whereas aCPP is associated with an increased risk of recurrence mainly in older children (≥ 3 years) and adults [9, 10] and in toddlers with pediatric B methylation subclass [11]. In contrast, the prognosis of CPC is poor and most pediatric survivors exhibit long-term cognitive and developmental deficits [5, 12].

Resection is the mainstay of treatment for any grade. However, surgery can cause complications impacting clinical status and outcomes. No preoperative findings have been identified that reliably predict the onset of postoperative hydrocephalus [6].

Adjuvant treatment can be considered for grade 2 and 3 CPT, including chemotherapy (CHT), specifically efficient in the pediatric setting. Radiotherapy (RT) can be considered in the adulthood or in pediatric patients when disease persistence is evident after CHT [13, 14].

The outcome is mainly related to CPT histological grade, but also to tumor size, location, and dissemination [13].

On gene expression level, unsupervised clustering segregates CPC from most CPP and aCPP [15, 16] whereas epigenetic profiling, using high-density DNA methylation array, divide CPT into three distinct DNA methylation subgroups: supratentorial pediatric low-risk CPT (CPP and aCPP; “pediatric A”), supratentorial pediatric high-risk choroid plexus tumors (CPP, aCPP and CPC; “pediatric B”) and infratentorial low-risk choroid plexus tumors in adults (CPP and aCPP, “adult”) [17].

In the context of genetic alterations involved in the tumorigenesis of CPT, investigations had shown that CPC mainly exhibits widespread chromosomal losses [18]. In addition, Thomas recently described the lack of recurrent driver alterations except for TP53 in pediatric CPT, whereas TERT promoter mutations or rarely a CCDC47-PRKCA fusion transcript, have been described in adults and associated with an aggressive clinical course [19].

Based on the rarity of such entity in adulthood, we presented our mono-institutional experience of 24 consecutive adult patients with CPT from 2003 to 2023: we highlighted tumor characteristics (including gene alterations such as TP53 and TERTp), the therapeutic approaches and the post-surgical evolution.

## Methods

This retrospective observational study collected clinical and radiological data from 24 consecutive CPT patients. All patients underwent surgery for CPT at our institution between January 2003 and May 2023 and were followed-up till May 2024.

We recorded information on symptoms at onset, radiological features, histo-molecular features, and treatments performed. Tumor characteristics, such as tumor location, enhancing lesion at computer tomography (CT) or magnetic resonance (MR), evidence of dissemination, and histopathology were also recorded. Radiological assessments included pre-surgical brain imaging (CT and MR with contrast enhancement). All radiological features were evaluated by two independent experienced neuro-radiologists blinded to the patient’s clinical outcomes. The extent of resection (EOR) was confirmed by comparison of preoperative and postoperative contrast-enhanced MR. EOR was categorized as gross-total resection (GTR) if 100% of the tumor was removed, subtotal resection (STR) if it was between 99% and 85%, and partial resection (PR) if it was less than 85%.

Histological diagnosis of CPT was made by a local neuropathologist, according to WHO CNS5 criteria.

Perioperative data included postoperative complications, length of hospitalization (LOS), intensive care unit stay, and days from resection to discharge. Medical and surgical complications were graded according to the Clavien-Dindo Grading Classification system (CDG) [20]. Complications were defined as any deviation from the normal peri-operative course (CDG ≥ 1) [20].

Clinical, radiological and therapy information was obtained from imaging reports, operative notes, hospital records, tumor registry, follow-up visits and/or telephone interviews.

Survival outcomes were assessed by Progression Free Survival (PFS), defined as the time from histological diagnosis to progression, and Overall survival (OS), the time to death or to the end of the study if the patient was still alive.

### Ethical standard and consents

The study was conducted according to the ethical rules for retrospective observational studies and was approved by the ethical committee of the Fondazione IRCCS Istituto Neurologico Carlo Besta (CET 19/24, 8th Apr 2024).

### Molecular analysis

Tumor samples were fixed in Carnoy and embedded in paraffin. Genomic DNA was extracted according to a standard phenol-chloroform protocol. Only those tumor areas previously identified as proliferating by hematoxylin and eosin (H&E) staining were selected and drawn from paraffin blocks by a lancet.

The mutational status of TP53 was determined by NGS sequencing on the tumoral sample. Library preparation was carried out using the Ion AmpliSeq Library Kit 2.0 (Life Technologies), as previously described [21]. The target regions were sequenced at a 250X depth.

The TERT promoter (TERTp) was amplified using hTERT_For CAGCGCTGCCTGAAACTC and hTERT_Rev GTCCTGCCCCTTCACCTT (163 bp product); Sanger sequencing was performed using 3500 Dx Genetic Analyzers (Applied Biosystems) and analyzed with Chromas Lite program (Technelysium DNA Sequencing Software 2.1.1).

## Results

**Fig. 1:**
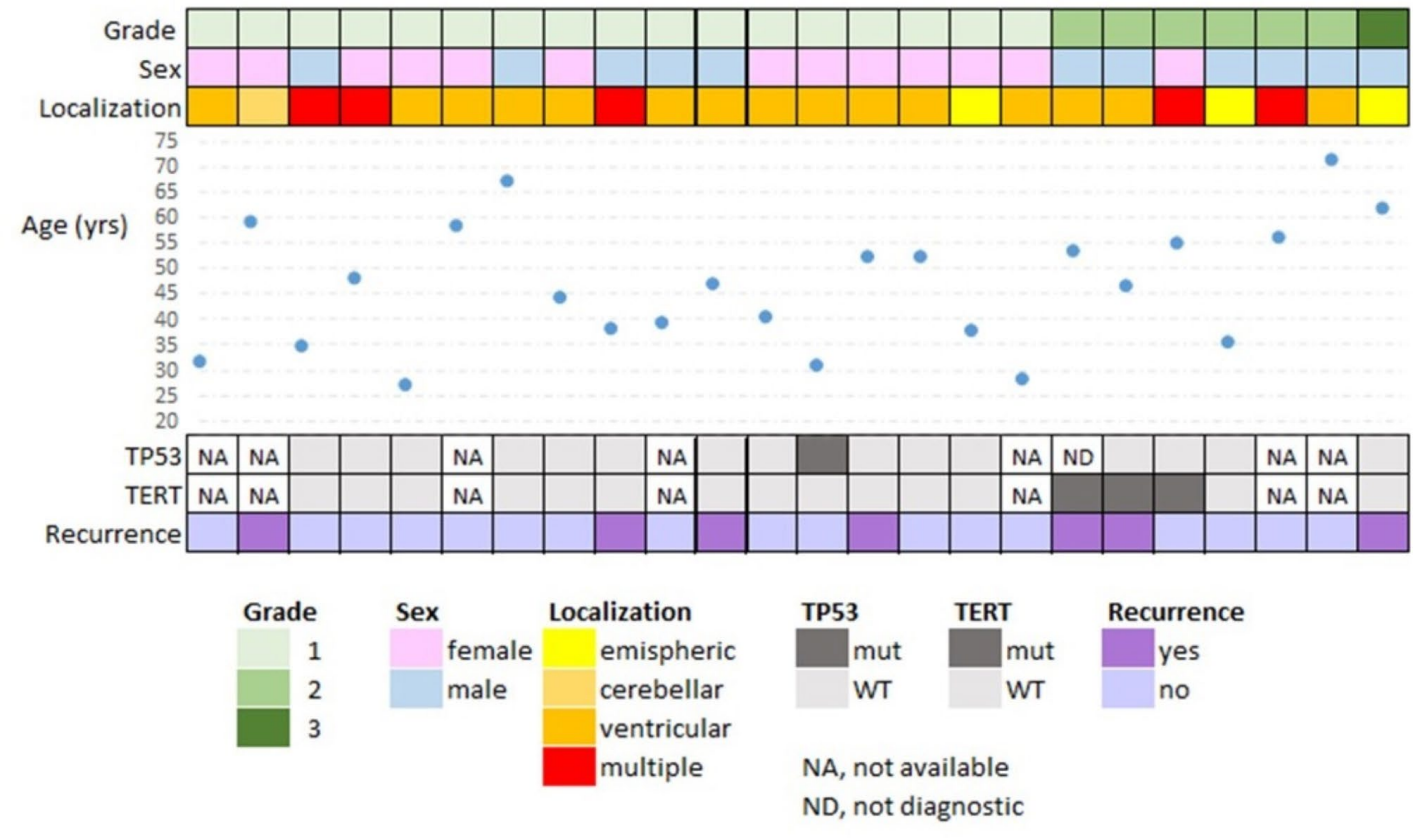
Descriptive and summary of radiological and histo-molecular features of all patients

We described 24 adult CPTs including details on demographic, clinic, imaging, and histological grade (Table 1; Fig. 1). Remarkably, in 16.7% (*n* = 4/24) CPTs were incidentally detected, as in the case of MR for unspecific headache or dizziness or due to a recent car accident. For the 20 symptomatic patients the median duration of symptoms before histological diagnosis was 29 weeks (from a few days to 7 years). Other co-morbidities included other tumors for four patients and demyelinating disease for one case. The extremely rare spinal presentation of an aCPT case in this series had already been published as a case report in a previous paper [22]. For all patients, a histological examination was performed (Fig. 1). TERTp mutation, present in17.6% of the cases was the canonical C228T; TP53 mutation was detected in 5.9%of the samples, and it was p.Cys176Phe c.527G > T.

**Table 1:** Demographics, clinics, radiology and histology of included patients

	*N* = 24
**Age at onset** (years) mean, (min-max)	45.14 (27.2–71.6)
**Oncological History**	4 (16.6%)
**KPS (median)**	90 (70–100)
Clinical signs *	
headache/vomiting (ICP)	8 (33.3%)
seizures	5 (20.8%)
cranial nerves	5 (20.8%)
cerebellar signs	11 (45.8%)
spinal	1 (4.2%)
no signs	4 (16.6%)
*Numbers exceed total because seven patients (29.2%) had more than one sign	
**Pre-surgery neuroimaging (brain and spine MR)**	
Site *	
ventricular	17 (70.8%)
hemispheric	4 (16.6%)
cerebellar	4 (16.6%)
spinal	1 (4.2%)
brainstem	2 (8.3%)
meningeal	1 (4.2%)
*Numbers exceed total because four patients (16.6%) had lesions in more than one site	
**Radiological evidence**	
hydrocephalus	1 (4.2%)
contrast enhancement	24 (100%)
**Post-surgical worsening neurological signs § (N)**	
headache/vomiting (ICP)	1
cortical-spinal tracts	3
cranial nerves	9
cerebellar signs	8
hemianopsia	1
§Numbers exceed total because some patients had more than one sign	

Regardless of histological grading, GTR, STR and PR were achieved in 17 (70.8%), 3 (12.5%) and 3 (12.5%) patients, respectively. For the remaining patient, a biopsy (*n* = 1, 4.2%) was performed. None of the cases underwent preoperative embolization.

76% of the cases (*n* = 16/21) experienced clinical worsening suddenly after surgery, as detailed in Table 1. The worsening was mostly transitory. In three cases no details about post-surgical clinical examination were available.

Medical complications were reported in 13 patients: hydrocephalus (*n* = 1), brain ischemia (*n* = 2), intraventricular hemorrhage with hygroma (*n* = 1), CSF fistula (*n* = 1), air embolism/pneumothorax/pneumomediastinum (*n* = 4), myocardial ischemia (*n* = 1), pneumonitis (*n* = 2), psychomotor agitation needing treatment (*n* = 2), severe anemiawith blood transfusion (*n* = 1), and severe hypotension treated with plasma expander (*n* = 2). In three cases, an external ventricular drain was temporarily inserted to improve CSF drainage: as planned in the surgical procedure in one case, to manage hydrocephalus and intraventricular hemorrhage in the other two. Four patients had more than one complication.

Based on CDG-rated complications (documented in *n* = 21/24), eight cases showed no complication. The remaining 14 patients got grade 1 (*n* = 5/14, 35.71%), grade 2 (*n* = 4/14, 28.57%), grade 3 (*n* = 2/14, 14.28%) and grade 4 (*n* = 2/14, 14.28%) at CDG scale.

The median period of hospitalization [length of stay (LOS)] and from surgery to discharge was 7 days (3–36) and 7 days (3–34) respectively. Admission in the intensive care unit ranged from 0 to 19 days, with a median of 1 day. 33% of patients needed intensive inpatient rehabilitation following the surgical hospitalization.

### Outcomes

By the end of the study, three patients had died (12.5%) and seven patients (25%) had a recurrence. One patient died 35 days after the second surgery at recurrence, for complications including intracranial hypertension, posterior inferior cerebellar artery ischemia, and meningoencephalitis.

**Fig. 2:**
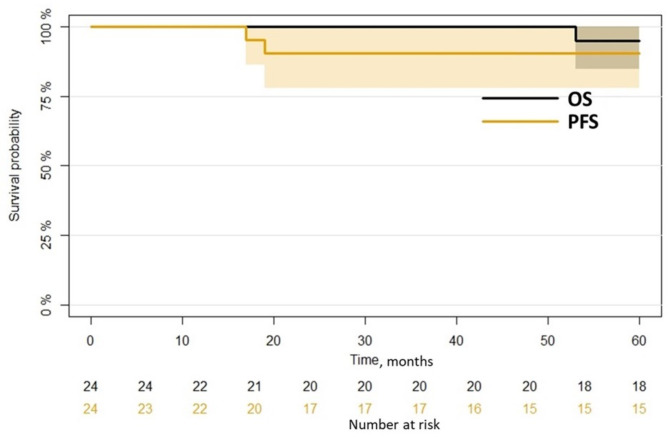
Kaplan mayer overall survival and progression free survival curves. Black line and black number at risk: OS; yellow line and numbers: PFS; colored area: confidence intervals

Median OS from surgery was 108 months IQR [63,143]. Median PFS was 90 months IQR [25, 136] (Fig. 2).

**Fig. 3:**
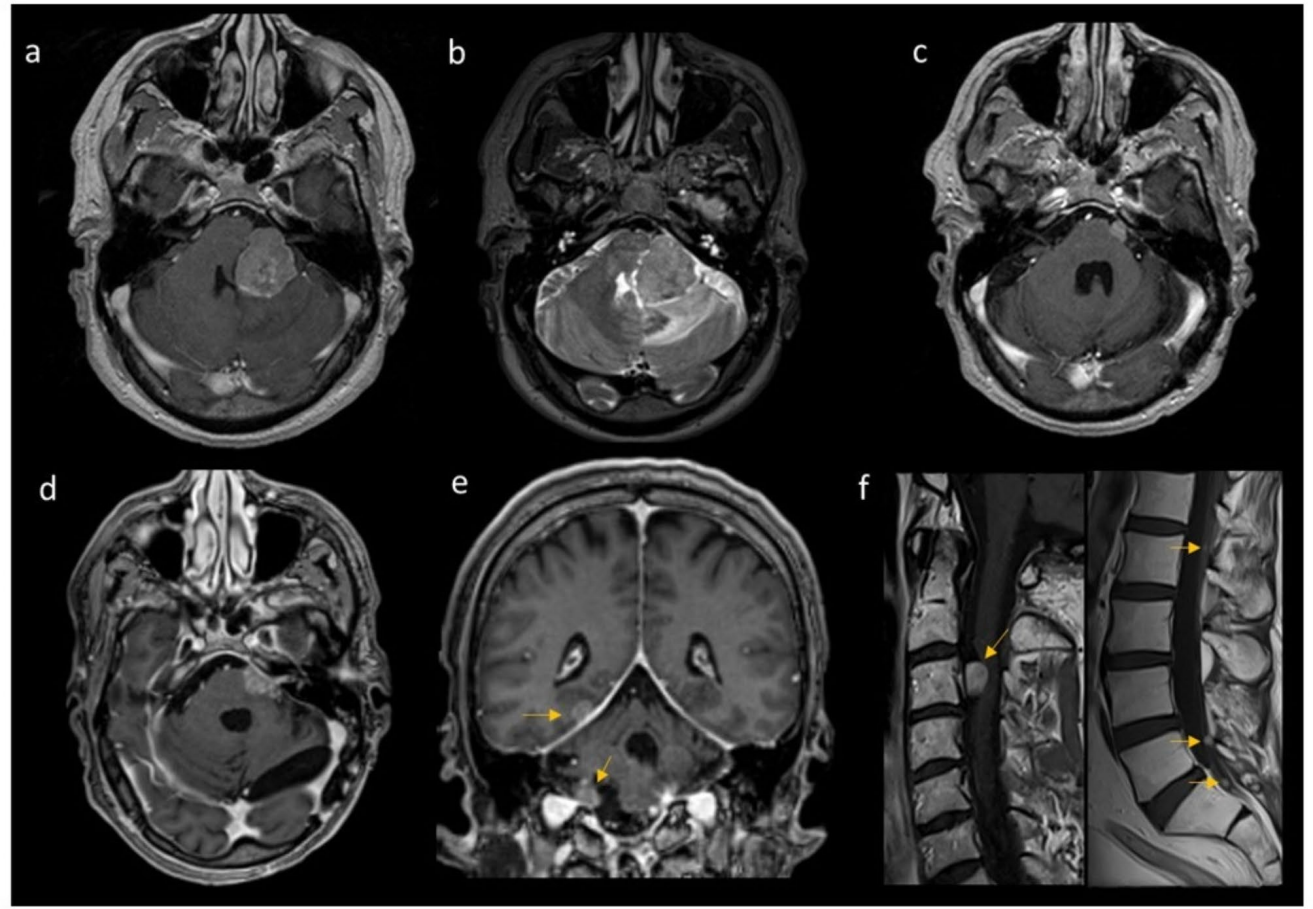
Patient 13; a 46-year-old man bearing an extra-axial cerebellopontine angle lesion with mass effect and reactive edema on cerebellar hemisphere, fourth ventricle and pons. First pre-surgical Magnetic Resonance (MR) (**a**, **b**); pre-radiotherapy MR 2 years later, at Disease Progression (**c**); last follow-up MR, 7 years from first surgery (**d**-**f**), showing supra-, infra-tentorial and spinal nodular dissemination (arrows). Brain (**a**-**e**) and spinal (**f**) T1-weighted images post- contrast agent administration (**a**, **c**-**f**) and T2-weighted images (**b**)

Adjuvant therapy was performed only for grade 2 or 3 tumors (overall 8.3% of the cases): the case of disseminated aCPT (Fig. 3) was treated with spinal conformational radiotherapy (RT) associated with radiosurgery, the grade 3 case got RT on cerebral lesion and spine. No cases were treated with CHT after the first diagnosis.

Seven cases had arecurrence. Exeresis was performed in three cases (one grade 1, one grade 2 and one grade 3), RT in two cases (one grade 1 and one grade 2) and one grade 3 recurrent patient had CHT after the second surgery. One case received an indication for clinical and radiological follow-up due to the very small size of recurrence with no new neurological signs.

In particular about the radiotherapy: one patients suffering from a disseminated disease at the diagnosis time had primary radiosurgery for 2 brain nodular locations (25 Gy/5 fractions) and whole spine irradiation with conventional fractionation (44 Gy/22 fractions) [22, Fig. 3]; 3 patients underwent radiosurgery (13–25 Gy in 1 to 5 fractions) for brain nodular recurrences and one out of these 3 had a whole spine irradiation with conventional fractionation (44 Gy/22 fractions) after the disease spreading, and finally one patients suffering from a WHO grade 3 plexus carcinoma had a 60 Gy adjuvant adrotherapy.

For the grade 3 CPT patient, CHT included temozolomide 5/28 at first recurrence, and further carboplatin/paclitaxel (patient 19). Moreover, for radionecrosis (at fourth recurrence), one grade 2 patient was treated with bevacizumab (patient 12).

## Discussion

Our adult series of 24 histologically proven CPT is much larger than what is reported up to now: Bahar et al. described 9 adult cases in 30 cases overall, and Hosmann et al. 19 cases out of 36 reported [12, 22]. Only Thomas 2021 described 28 adult cases included in a cohort of 47 overall cases, addressing genetic profile but lacking clinical, peri-surgical, and therapeutic data information [18].

The median age at onset was 45 years, and the sex ratio was similar to the adult sub-cohort by Hosmann [12]. Incidental detection (17% in our series) was consistent with other reports [23, 24].

In our series, most cases were located at the fourth ventricle and rarely there was radiological pre-surgical hydrocephalus differently from a previous description [25]. Symptoms associated with intracranial hypertension were present in 33% of our patients, less frequently than reported in literature often relative to the pediatric population [8].

CPT in adults may be surgically removed through either endoscopic transventricular or craniotomic approaches. The choice of the specific approach depends on tumor location, size, possible histological grade, and associated CSF disturbances. The ventriculoperitoneal shunt may be reasonable in cases of chronic hydrocephalus associated with very slow-growing lesions.

GTR of CPT lesions remains crucial. In our series, we reached at least STR in 83% of the cases.

We documented post-surgical worsening in a large part of the cohort (76%). Complications described in the literature were very variable [13, 23], often lacking specific definitions or even unreported.

We used a validated and reproducible scale to grade worsening and collected reliable information on peri-operative clinical problems.

Other types of postoperative complications have been reported: CSF leak (10.9%), infection (8.7%), new weakness (6.5%), seizures (4.3%), stroke (4.3%), hemorrhage (2.2%), and sigmoid sinus vein thrombosis (2.2%) were noted in a cohort of 46 any-age patients [26]. We were not able to summarize these data as multiple complication could refer to the same patient [26]. They also reported a higher need for (at least 1) postoperative hydrocephalus surgery compared to our data (24% vs. 6.25% respectively) [26].

Surgery is the standard initial treatment for CPT, but the features of the rich vascular supply and the deep intraventricular CPT location should be taken into account. Based on serious cases of post-operative complications, the counterpoint could be biopsy followed by neoadjuvant CHT in order to reduce the risk of bleeding and less morbidity in the post-operative period, as described in studies such as Schneider et al., 2015 [27] and Lafay-Cousin et al., 2010 [28].

Non-GTR was described as a negative prognostic factor in CPT [14].

Histology grading remains a significant predictor of OS [3]. In our study, due to the small number of grade 2–3 CPT patients, we could not present any statement about it.

Regarding the molecular profile, we detected TERTp mutation (C228T) in 17.6% of the subjects. Thomas and co-authors [19] documented the same C228T *TERT* promoter mutation with similar frequency (16%); in addition, they reported also one case of C250T TERTp mutation (2%) and one case of uncertain significance.

In our series, one case harbored somatic TP53 mutation and, although 20.8% (*n* = 5/24) of patients had oncological history, none were suggestive of Li-Fraumeni Syndrome.

The identification of prognostic genetic alterations could result in immediate clinical implications, as suggested for TP53 mutations in childhood CPT [5].

## Conclusion

We confirm that CPT can occur in adults and are mostly grade 1 tumors located in the ventricular system.

TERT, TP53 and GTR were possible predictive factors of longer PFS in adult CPT. Gene mutations were present in our series only in grade 2 or grade 3 patients. We attempted to verify these assumptions by analyzing our data, but the limited number of events prevented any useful comparison.

The use of adjuvant therapy is still under discussion, and molecular patterns will have to be included in the near future to provide adequate individual treatment by a multidisciplinary approach. Surgical therapy is the gold standard, but biopsy and neo-adjuvant CHT should be considered to reduce perioperative morbidity and sequelae, especially when approaching grade 1 tumors.

Overall, in such an infrequent disease, retrospective analyses, combined with expert opinion can represent the ‘background information’ and carry weight in decision-making. This extremely rare entity will need to be studied with a collaborative effort in international studies. The proper number of adult cases will enable the oncologist network to verify our preliminary observations.

## Data Availability

Data will be available at: https://zenodo.org/communities/besta/.

## Abbreviations

KPS: Karnofsky performance status scale
ICP: Intracranial pressure
MR: Magnetic Resonance
OS: Overall Survival
PFS: Progression Free Survival
